# Incidence Analyses and Space-Time Cluster Detection of Hepatitis C in Fujian Province of China from 2006 to 2010

**DOI:** 10.1371/journal.pone.0040872

**Published:** 2012-07-19

**Authors:** Shunquan Wu, Fuquan Wu, Rongtao Hong, Jia He

**Affiliations:** 1 Department of Health Statistics, Second Military Medical University, Shanghai, China; 2 International Cooperation Laboratory on Signal Transduction, Eastern Hepatobiliary Surgery Institute, Second Military Medical University, Shanghai, China; 3 Fujian Center for Disease Control and Prevetion, Fuzhou, Fujian Province of China; University of Modena & Reggio Emilia, Italy

## Abstract

**Background:**

There is limited epidemiologic information about the incidence of hepatitis C in China, and few studies have applied space-time scan statistic to detect clusters of hepatitis C and made adjustment for temporal trend and relative risk of regions.

**Methodology and Principal Findings:**

We analyzed the temporal changes and characteristics of incidence of hepatitis C in Fujian Province from 2006 through 2010. The discrete Poisson model of space-time scan statistic was chosen for cluster detection. Data on new cases of hepatitis C were obtained from the Center for Disease Control and Prevention of Fujian Province. Between 2006 and 2010, there was an annualized increase in the incidence of hepatitis C of 23.0 percent, from 928 cases (2.63 per 100,000 persons) to 2,180 cases (6.01 per 100,000 persons). The incidence among women increased more rapidly. The cumulative incidence showed that people who were over 60 years had the highest risk to suffer hepatitis C (52.51 per 100,000 persons), and women had lower risk compared to men (OR = 0.69). Putian had the highest cumulative incidence among all the regions (86.95 per 100,000 persons). The most likely cluster was identified in Putian during March to August in 2009 without adjustment, but it shifted to three contiguous cities with a two-month duration after adjustment for temporal trend and relative risk of regions.

**Conclusions/Significance:**

The incidence of hepatitis C is increasing in Fujian Province, and women are at a more rapid pace. The space-time scan statistic is useful as a screening tool for clusters of hepatitis C, with adjustment for temporal trend and relative risk of regions recommended.

## Introduction

Hepatitis C has become a significant public health burden in both industrialized and developing countries. It is spread by hepatitis C virus (HCV) and may lead to cirrhosis, hepatocellular carcinoma and liver transplantation [1], [2]. An estimated 170 million persons worldwide have chronic infection and 3 to 4 million new cases occur each year [3]–[5]. There is a wide range of prevalence of hepatitis C in China, whose citizens account for one-fifth of the world’s population. HCV seroprevalence in the general population ranges from 3.2% to 4.1% in China [3], [6], [7]. Hepatitis C control and prevention has become a great challenge for both China and all over the world.

Most of the morbidity associated with hepatitis C is realized through the development of chronic liver disease years after initial acquisition of the infection. Thus, the past and present incidence of hepatitis C is a major determinant of the future burden of the disease [8], [9]. However, there is limited epidemiologic information about the temporal changes in hepatitis C incidence in China and little is known about the characteristics of the new cases.

Furthermore, early detection of outbreaks enables public health officials to implement measures to control the disease before it becomes widespread at the earliest possible time. Space-time cluster detection is an important tool in hepatitis C surveillance to identify various size, areas and duration of elevated risk. Space-time scan statistic proposed by Kulldorff is becoming popular in detecting space-time clusters in various diseases [10], including infectious diseases [11], [12] and cancers [13], [14], and it has proven useful as a tool for identifying cluster alarms that are not likely to be of public health importance. It is done by gradually scanning a window across time and space, noting the number of observed and expected observations inside the window at each location. A software package of SaTScan is freely available to apply this statistical approach [15]. The approach of space-time scan statistic has not yet been widely used in China, and few studies have applied it on hepatitis.

In the present study, we collected data of newly diagnosed cases of hepatitis C in Fujian Province, which is located in southeast of China bordering the East China Sea, from 2006 to 2010. We sought to determine the incidence trends of hepatitis C and provide characteristics of it for use in epidemiologic estimates, according to the newly diagnosed cases. Another purpose of this study was to detect the space-time clusters of hepatitis C in Fujian Province by using space-time scan statistic, and to prove that it is an effective approach for hepatitis C cluster detection.

## Methods

### Data Source

The data of newly diagnosed cases of hepatitis C were obtained from the Center for Disease Control and Prevention (CDC) of Fujian Province. China built the world’s largest internet-based disease reporting system, called the China Information System for Disease Control and Prevention (CISDCP) in 2004 after the SARS epidemic [16], and since then, cases of infectious diseases when initially diagnosed are reported to local CDC by medical institutions at all levels. And after checking the records,CDC saves the data to CISDCP. The CISDCP is a real-time system being updated daily. The data in CISDCP contain the patients’ basic personal information,including name, age, sex, habitual residence, time of onset of the disease and other information. The current study covers all reported cases of hepatitis C in Fujian with the onset time ranges from Jan 1, 2006 to Dec 31, 2010. The estimated population in each year in Fujian during the 2006–2010 period, along with age-, sex- and region-specific population data were also obtained. The study was approved by the Second Military Medical University Ethics Committee. All patients had signed a written informed consent before the information was stored into the database.

### Statistical Methods

#### Incidence analyses

We calculated the number of newly diagnosed cases of hepatitis C alone, or in different age groups and sexes. The incidence of hepatitis C (per 100,000 persons) in each year and cumulative incidences (per 100,000 persons) of five years among different variables (time of onset, age at onset, sex, and geographic region) were also calculated. SAS 9.1.3 was used, and the odds ratio (OR) and 95% confidence interval (CI) were estimated by conducting the Chi-square test to assess the trends. A p-value of less than 0.05 was considered to be statistically significant. The characteristics of new cases and the temporal trends of incidence were reported.

#### Cluster analyses

The space-time scan statistic is defined by a cylindrical window with a geographic base and with height corresponding to time. The window is moved in the study region with continuously varying bases and heights. In effect, an infinite number of overlapping cylinders of different size and shape were obtained, and each one reflects a possible cluster. The numbers of disease cases inside and outside each cylinder are noted, together with the expected number of cases. The likelihood is calculated for each cylinder according to these numbers [10]. The cylinder with the maximum likelihood ratio is denoted the most likely cluster. A limit number of secondary clusters are also identified and will be ordered in descending order by the likelihood ratios. The p-value is obtained through Monte Carlo hypothesis testing [17].

In this study, we used SaTScan software [15], which is based on space-time scan statistic theory, to detect the space-time clusters of hepatitis C in Fujian Province during 2006 to 2010. The discrete Poisson model was used as cases of hepatitis C was assumed to be Poisson distributed with constant risk over space and time under the null hypothesis, and with different risk inside and outside at least one cylinder under the alternative hypothesis. The specific statistical theory behind the space-time scan statistics used in the SaTScan software is described in detail by Kulldorff for discrete Poisson model [10].

If there was an increasing/decreasing temporal trend in hepatitis C cases, the space-time scan statistics would pick up that trend by assigning a cluster during the end/beginning of the study period. And if certain regions had higher relative risk (RR) of hepatitis C cases, these regions might be more likely to be identified as clusters. So we conducted two separate cluster detection analyses, one with no adjustment and the other with adjustment for temporal trend and RR of regions. As the developer of space-time scan statistics states, the best way to adjust for a temporal trend is by specifying the percent yearly increase or decrease in the rate that is to be adjusted for [15]. So we made a temporal trend adjustment by setting an annual growth rate of incidence of the data being analyzed to the SaTScan software and using a log linear trend adjustment. RR of regions was adjusted by writing an adjustment file that included the RRs of incidence in different regions as calculated in this study, and the file was imported to the SaTScan software.

To run the SaTScan software, we wrote a SAS program that generated the case file. The population file was made according to the population data of Fujian Province, and the coordinates file was made according to the latitude and longitude coordinates of 9 district cities in Fujian Province. Retrospective space-time analysis was selected as we used historic data. The maximum spatial cluster size was set to 50% of the population at risk as recommended, and the maximum temporal cluster size was set to 6 months. This meant that the evaluation would include outbreaks with a circle radius size anywhere between zero and 50% population at risk, and a time length (cylinder height) of 1 to 6 months. In order to obtain more precise null occurrence rates, we set the number of Monte Carlo replications to 9,999, which meant that the smallest p-value we could get was 0.0001.

We reported the most likely clusters of both with no adjustment used and with temporal trend and RR of regions adjusted, as well as the secondary clusters ordered in descending order by the likelihood ratios. Signals of the space-time clusters we detected after adjustment for temporal trend and RR of regions were examined.

## Results

### Demographic Characteristics

During the study period, the average population was 35,867,130 in Fujian Province, with an approximately 0.6% increase per year. The mean ages from 2006 to 2010 were 32.6, 32.8, 33.0, 33.2 and 33.5 years, respectively, and the average proportion of males to females was 1.04∶1.

### Case Patterns, Age and Sex Distribution

**Figure 1:**
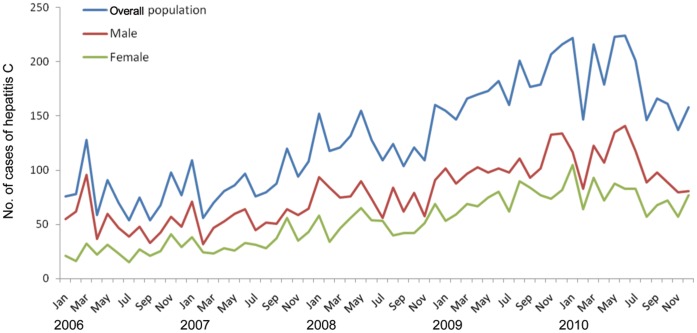
Number of cases of hepatitis C in Fujian Province of China, from 2006 to 2010.

There were a total of 7839 reported cases in Fujian Province during Jan 1, 2006 to Dec 31, 2010, with approximate 60% were male. The number of newly diagnosed patients with hepatitis C per year increased from 928 in 2006 to 2,180 in 2010, a 135% rise (an increase of 23.8% per year). Figure 1 describes the overall population of hepatitis C cases reported during the study period, as well as among male and female population. Men had a higher level of occurrences than women in the whole period. As a whole, the cases showed an increasing trend though went up and down unregularly, and there was a similar pattern among men and women.

**Figure 2:**
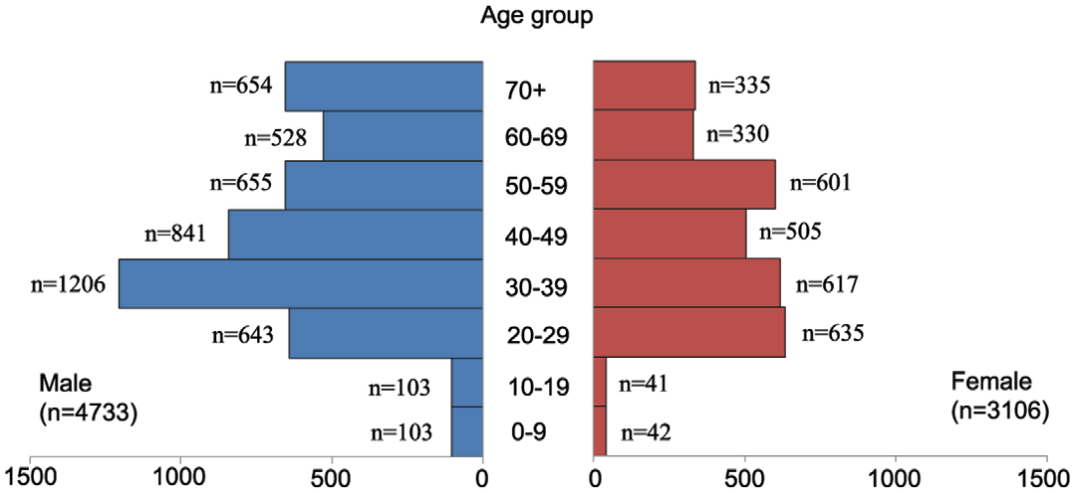
Age and sex distribution of hepatitis C in Fujian Province of China, from 2006 to 2010.

The mean age of patients was 45.4 years (95% CI, 45.0 to 45.8 years) with persistent increase from 2006 (43.5 years, 95% CI, 42.3 to 44.7 years) to 2009 (46.4 years, 95% CI, 45.6 to 47.1 years), and a little drop in 2010 (45.7 years, 95% CI, 45.0 to 46.5 years). Figure 2 provides information of hepatitis C cases distributed among different age groups and sexes. More than two thirds of patients were 20 to 59 years (5,703, 72.8%), and men had more cases than women among all age groups.

### Incidence

**Figure 3:**
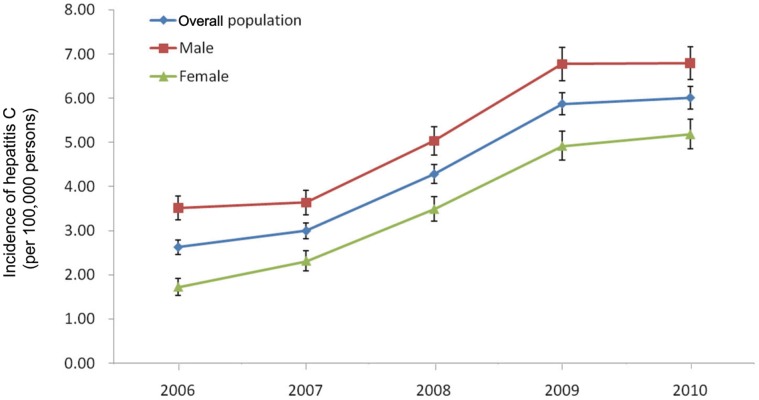
Incidence of hepatitis C in Fujian Province of China, from 2006 to 2010. Points represent the number of cases per 100,000 persons, and error bars the 95% confidence interval.

The incidences of hepatitis C in Fujian Province from 2006 to 2010 are shown in Figure 3. The number of reported hepatitis C cases per 100,000 persons has progressively increased in overall population, from 2.63 (95% CI, 2.46 to 2.79) in 2006 to 6.01 (95% CI, 5.76 to 6.26) in 2010, an increase of 129% (approximately an increase of 23.0% per year). There was a similar pattern to the increases in incidence among men and among women. The number of cases per 100,000 persons among men was much higher than that among women during the study period, 3.52 (95% CI, 3.24 to 3.79) to 6.80 (95% CI, 6.42 to 7.17) *vs.* 1.72 (95% CI, 1.53 to 1.92) to 5.19 (95% CI, 4.85 to 5.52). However, the incidence among women increased more rapidly (an annualized increase of 31.7% *vs.* 17.9%). The increasing trend was most apparent during the middle of the period, from 2007 through 2009. In addition, we also estimated the OR of increasing incidences from 2006 to 2010, which are shown in Table 1. Consistent results were obtained that OR increased as time went by and women had a higher OR. When comparing incidence in 2010 to that in 2006, the OR was 3.01 (95% CI, 2.64 to 3.43) among women and 1.93 (95% CI, 1.76 to 2.13) among men, while that was 2.29 (95% CI, 2.12 to 2.47) in overall population.

**Table 1:** Odds ratios of increasing incidences of hepatitis C in Fujian Province from 2006 to 2010.

Year	Whole population		Male		Female	
	OR (95%CI)	P-Value	OR(95%CI)	P-Value	OR(95%CI)	P-Value
2006	1.00[Reference]		1.00[Reference]		1.00[Reference]	
2007	1.14(1.04–1.25)	0.003	1.03(0.93–1.15)	0.54	1.34(1.16–1.56)	<0.001
2008	1.63(1.50–1.77)	<0.001	1.43(1.29–1.58)	<0.001	2.02(1.76–2.32)	<0.001
2009	2.24(2.07–2.42)	<0.001	1.93(1.75–2.12)	<0.001	2.86(2.51–3.25)	<0.001
2010	2.29(2.12–2.47)	<0.001	1.93(1.76–2.13)	<0.001	3.01(2.64–3.43)	<0.001

Cumulative incidences and ORs were calculated among different variables, as Table 2 shows, to assess the relative risks of incidence in different seasons, ages, sexes, and geographic regions. The cumulative incidence of hepatitis C was the lowest between July and September (5.17 per 100,000 persons), and the OR was 0.93 (95% CI, 0.87 to 0.99) referenced to the baseline. Regarding to age distribution, old people were more vulnerable to suffer hepatitis C than young people, and the cumulative incidence was 52.51 per 100,000 persons in people who were over 60 years, and when referenced to the baseline, the OR was 5.65 (95% CI, 5.28 to 6.04). Hepatitis C was rare before age 30 years. The cumulative incidence was 18.12 per 100,000 persons among women and 26.29 per 100,000 persons among men (OR = 0.69, 95% CI, 0.66 to 0.72). The occurrence rate of hepatitis C was not evenly distributed in different regions of Fujian Province. Of the 9 district cities, Putian (in east central Fujian) had the highest cumulative incidence (86.95 per 100,000 persons), and the OR was 2.96 when referenced to Fuzhou, the capital city, with 95% CI, 2.79 to 3.14. Zhangzhou (in southern Fujian) had the lowest cumulative incidence (5.54 per 100,000 persons), which was one-fifteenth of that in Putian. Further exploration found that the proportion of patients lived in rural areas in Putian was significantly higher than that in overall Fujian Province (72.3% *vs.* 33.6%, χ^2^ = 1142.5, p<0.001).

**Table 2:** Cumulative incidences and odds ratios of hepatitis C in Fujian Province among diff erent variables, from 2006 to 2010 (n = 35139825).

Variable	Cumulative incidence(per 100,000 persons)	OR(95%CI)	P-Value
Time of onset			
Jan–Mar	5.58	1.00[Reference]	
Apr–Jun	5.83	1.05(0.98–1.11)	0.16
Jul–Sep	5.17	0.93(0.87–0.99)	0.02
Oct–Dec	5.73	1.03(0.96–1.09)	0.41
Age at onset, y			
0–29	9.31	1.00[Reference]	
30–59	29.93	3.22(3.04–3.41)	<0.001
60 and above	52.51	5.65(5.28–6.04)	<0.001
Sex			
Male	26.29	1.00[Reference]	
Female	18.12	0.69(0.66–0.72)	<0.001
Geographic region			
Fuzhou	29.37	1.00[Reference]	
Xiamen	29.45	1.00(0.92–1.09)	0.95
Putian	86.95	2.96(2.79–3.14)	<0.001
Sanming	16.07	0.55(0.49–0.61)	<0.001
Quanzhou	12.03	0.41(0.38–0.44)	<0.001
Zhangzhou	5.54	0.19(0.17–0.21)	<0.001
Nanping	8.82	0.30(0.26–0.34)	<0.001
Longyan	12.96	0.44(0.39–0.49)	<0.001
Ningde	18.09	0.62(0.56–0.68)	<0.001

### Space-time Clusters

The space-time clusters found by SaTScan using discrete Poisson model are displayed in Table 3. Without adjustment, the most likely cluster was located in Putian with the duration ranged from March to August in 2009. In all, 383 cases were reported (61.43 expected) and the cluster had a relative risk (RR) of 6.50. Two statistically significant secondary clusters were identified, one was composed of three contiguous cities (Nanping, Sanming, and Fuzhou) with the duration ranged from November, 2009 to March, 2010, and the other was located in Xiamen with duration ranged from June to September in 2010.

**Table 3:** Retrospective space-time analysis of hepatitis C, using the discrete Poisson model of space-time statistic.

	Time frame	Population	No. of cases	Expected cases	RR*	LLR**	P-value
**No adjustment**							
Most likely cluster: Putian	Mar, 2009–Aug, 2009	2945364	383	61.43	6.50	386.16	<0.0001
Secondary cluster: Nanping, Sanming, Fuzhou	Nov, 2009–Mar, 2010	12267879	408	220.88	1.89	65.57	<0.0001
Secondary cluster: Xiamen	Jun, 2010–Sep, 2010	2425558	65	36.38	1.79	9.16	0.0388
**Adjustment for temporal trend** a **and RR of regions**							
Most likely cluster: Nanping, Sanming, Fuzhou	Nov, 2009–Dec, 2009	12267879	208	110.38	1.91	34.80	<0.0001
Secondary cluster: Ningde	May, 2010–Jul, 2010	3133469	93	40.24	2.33	25.33	<0.0001
Secondary cluster: Putian	Feb, 2009–Jun, 2009	2945364	323	218.36	1.50	22.54	<0.0001
Secondary cluster: Quanzhou	Aug, 2009–Oct, 2009	7770465	97	56.62	1.72	11.94	0.0022

* RR: Relative risk.

** LLR: Log likelihood ratio.

a Incidence increased by approximately 23.0% per year.

As an increasing temporal trend in hepatitis C cases and inconsistent risks in different regions existed, we ran the SaTScan once again by adjusting the temporal trend and RR of geographic regions. The strongest signal was a two-month cluster that began in November and continued to December in 2009, covering Naping, Sanming, and Fuzhou. This signal had 208 cases observed when 110.38 were expected (RR = 1.91). Hence, the increasing temporal trend and the higher RR of Putian might partly explain the most likely cluster in the first analysis. There were three statistically significant secondary clusters followed. The first one was in Ningde which began in May, 2010 and extended into July of that year. The remaining two were in Putian and Quanzhou, and lasted from February to June and August to October in 2009, respectively.

**Figure 4:**
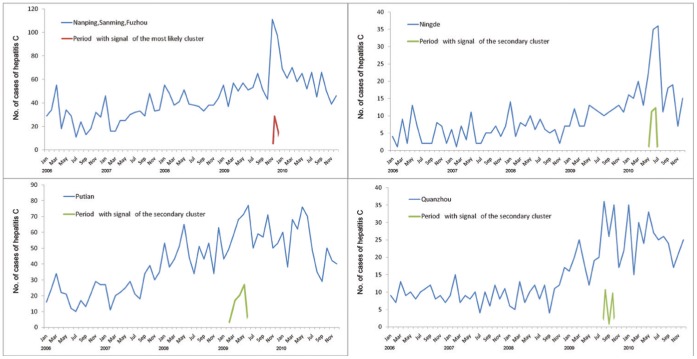
The number of cases of hepatitis C in regions identified as the clusters after adjustment for temporal trend and relative risk of regions, with the signal duration marked.

We examined the adjusted cluster signals by observing the number of cases in the regions included in each cluster separately. As Figure 4 shows, the number of cases in Nanping, sanming, and Fuzhou had a sharp increase between November and December in 2009, the period with the signal of the most likely cluster. Likewise, the number of cases in Ningde peaked during May to July in 2010. And in both Putian and Quanzhou, the signals of secondary cluster immediately preceded the increases of hepatitis C cases.

## Discussion

Although it is well known that hepatitis C is common in China, there is little information about its incidence, especially in recent years. Given the current uncertainty, we chose Fujian Province as a representative of China to analyze the incidence of hepatitis C between 2006 and 2010.

We observed a doubling of new cases of hepatitis C with a steady increase during this period in Fujian, and the incidence of hepatitis C has significantly increased. Compared to 2006, people were significantly more likely to be infected in 2010, assessed by the OR. The study of changes in hepatitis C incidence is important for several reasons. An increase in incidence over time may lead to hypotheses concerning sources of HCV transmission. Identification of such sources is of importance in reducing exposure to hazardous factors and thereby the risk of hepatitis C infection. The increasing incidence of hepatitis C also suggests that the growing need for such care is becoming a more important public health issue.

Demographic differences in the incidence of hepatitis C existed during the five-year study period. Men were more susceptible to hepatitis C who had much more cases and higher incidence than women, and this was supported by previous studies [18], [19]. Despite the fact that men were consistently more likely to have hepatitis C, greater increase in incidence occurred among women. Senior citizens over 60 years got the highest incidence, though the most cases of hepatitis C occurred among young and adults. The prevalence of hepatitis C showed a typical pattern of old people and young adults. Reasons for this pattern were unclear. This may be related to the following two factors: firstly, senior citizens over 60 years might have the history of blood transfusion, for blood transfusion is a major cause of the spread of HCV [20]–[22]; secondly, the major source of employment for the community comes from young adults whose occupations are widely distributed, and they contact with a large range of people and have more chances of infection. Special care should be taken to that two population groups. Moreover, the mean age of Fujian population has increased gradually, which makes us assume the increase of incidence of hepatitis C is associated with the increasing age, since the risk of infection was elevated with the age growth as identified in this study. Besides, the apparent regional disparities are worthy of being noted. Putian, Xiamen, and Fuzhou had relatively higher cumulative incidence, compared to other regions. The prominent risk in Putian was extremely striking. The five-year cumulative incidence in Putian was 86.95 per 100,000 persons, which was over 15 times as high as that in Zhangzhou. A large proportion of peasant-patients suggests that HCV spread is not well controlled in the rural areas in Putian, and the environment there may serve as a reservoir for infectious virus. However, other explanations for the high incidence of hepatitis C in Putian should be further investigated and effective intervention programs must be performed.

Hepatitis C is the second most common cause of chronic liver disease and hepatocellular carcinoma after hepatitis B [5]. Considering the present unavailability of vaccine and the lack of effective therapy, the setting up of control actions targeting specific zones and periods is therefore a priority, and space-time cluster detection is of utmost importance.

In this study, the space-time scan statistic identified the locations and durations of the most likely clusters as well as the secondary clusters. Putian was identified as the most likely cluster before adjustment for temporal trend and RR of regions, using the discrete Poisson model of space-time scan statistics, and this cluster coincided with the highest incidence of hepatitis C in Putian. After the adjustment, it became one of the secondary clusters and the time frame was shortened. We can conclude that the increasing cases at the posterior study period and the remarkably higher risk of incidence in Putian concealed a more apparent outbreak among three contiguous cities of Nanping, Sanmig, and Fuzhou, as Figure 4 shows. Similar outbreak was also found in Ningde, the region identified as a secondary cluster after the adjustment where borders Nanping and Fuzhou. The proximity of the two clusters indicates similar transmission may exist in those adjacent regions. The different results obtained before and after adjustment suggest that adjustment for temporal trend and RR of regions is needed when using space-time scan statistic for outbreak detection, because this would make it feasible to investigate all ‘unusual’ events, meaningfully increasing the value of the surveillance.

To our best knowledge, this is the first research that attempts to use space-time scan statistics to detect clusters of hepatitis C and makes adjustment for temporal trend and RR of regions. Space-time permutation model of space-time scan statistic can also be used for cluster detection, but it only uses case data and cannot distinguish whether the cluster is due to an increased risk of disease or to a sudden increase of population [15]. Thus, the discrete Poisson model, as used in this study, is a better choice which utilizes the extra population information. There were also several other methods used for disease outbreaks detection [23]–[26], but these methods are all purely spatial or temporal, and rarely has a spatio-temporal statistical model been used. Though China has recognized that surveillance and detection of disease outbreaks is critical, sophisticated statistical model has not yet been applied [16], [27]. We think space-time scan statistic is an ideal method for surveillance of hepatitis C outbreaks and should be added to the public health official’s toolbox in China. We recommend that discrete Poisson model to be used and temporal trend and RR of regions should be adjusted.

One limitation of this study is that we might underestimate the incidence of hepatitis C. One reason for this is that the acute hepatitis C is often clinically mild or completely asymptomatic and is rarely recognized [28], so the rate of missed diagnosis of hepatitis C is quite high. A second reason can be attributed to the immature disease surveillance system with a certain proportion of cases not reported to CISDCP; however, it is unavoidable since even in countries with well-established surveillance systems, the incidence of hepatitis C is also underestimated [29]–[31]. Another limitation is that the scan statistic uses a circular scanning window with variable size to identify the potential clusters, and it is difficult to detect non-circular clusters flexibly [32]. Despite these limitations, this study has important public health significances, for the results of the increasing risk of hepatitis C infection should be taken seriously, and it is the first step to apply the space-time scan statistic to the cluster detection of hepatitis C.

In conclusion, we analyzed the trends and characteristics of the incidence of hepatitis C in Fujian Province of China in recent years in this paper. We also proposed a space-time cluster detection using space-time scan statistic. We found that the incidence of hepatitis C is growing rapidly and is likely to increase substantially in the coming years. Special care should be taken to old people and young adults and also to the women. The space-time scan statistic is an effective method for cluster detection. However, temporal trend and RR of regions should be adjusted to obtain more reliable results. There is still a need for studies to confirm the root cause of the increasing incidence and outbreaks of hepatitis C, and the method of space-time scan statistic has much room for improvement.
